# Expression of Fap amyloids in *Pseudomonas aeruginosa*, *P. fluorescens*, and *P. putida* results in aggregation and increased biofilm formation

**DOI:** 10.1002/mbo3.81

**Published:** 2013-03-18

**Authors:** Morten S Dueholm, Mads T Søndergaard, Martin Nilsson, Gunna Christiansen, Allan Stensballe, Michael T Overgaard, Michael Givskov, Tim Tolker-Nielsen, Daniel E Otzen, Per H Nielsen

**Affiliations:** 1 Department of Biotechnology, Chemistry and Environmental Engineering, Aalborg UniversityAalborg, Denmark; 2 Department of International Health, Immunology and Microbiology, University of CopenhagenCopenhagen, Denmark; 3 Department of Biomedicine, Aarhus UniversityAarhus, Denmark; 4 Singapore Centre on Environmental Life Sciences Engineering, Nanyang Technological UniversitySingapore; 5 Interdisciplinary Nanoscience Center (iNANO), Centre for Insoluble Protein Structures (inSPIN), Department of Molecular Biology and Genetics, Aarhus UniversityAarhus C, Denmark

**Keywords:** Amyloids, biofilm, fap, FuBA, Pseudomonas

## Abstract

The *fap* operon, encoding functional amyloids in *Pseudomonas* (Fap), is present in most pseudomonads, but so far the expression and importance for biofilm formation has only been investigated for *P. fluorescens* strain UK4. In this study, we demonstrate the capacity of *P. aeruginosa* PAO1, *P. fluorescens* Pf-5, and *P. putida* F1 to express Fap fibrils, and investigated the effect of Fap expression on aggregation and biofilm formation. The *fap* operon in all three *Pseudomonas* species conferred the ability to express Fap fibrils as shown using a recombinant approach. This Fap overexpression consistently resulted in highly aggregative phenotypes and in increased biofilm formation. Detailed biophysical investigations of purified fibrils confirmed FapC as the main fibril monomer and supported the role of FapB as a minor, nucleating constituent as also indicated by bioinformatic analysis. Bioinformatics analysis suggested FapF and FapD as a potential β-barrel membrane pore and protease, respectively. Manipulation of the *fap* operon showed that FapA affects monomer composition of the final amyloid fibril, and that FapB is an amyloid protein, probably a nucleator for FapC polymerization. Our study highlights the *fap* operon as a molecular machine for functional amyloid formation.

## Introduction

Pseudomonads are, like most other bacteria, able to adhere to surfaces and form biofilms allowing the bacteria to stay in a protected environment. Biofilms formed by pseudomonads are of medical and technical importance, and substantial research has been carried out to elucidate the molecular mechanisms involved in *Pseudomonas* biofilm formation (Klausen et al. 2006). Many different extracellular components such as polysaccharides, proteins, and extracellular DNA (eDNA) are involved in adhesion and biofilm formation by the pseudomonads (Pamp et al. 2007). These components are generally well studied and their specific function fairly well understood (Jahn et al. 1999; Whitchurch et al. 2002; Matsukawa and Greenberg 2004; Allesen-Holm et al. 2006; Ma et al. 2009). Recently, however, we demonstrated that functional bacterial amyloids (FuBA) may play a role in *Pseudomonas* biofilm formation (Dueholm et al. 2010). FuBA are known to be of key importance in adhesion, biofilm formation, and virulence in *Escherichia coli* and *Salmonella*, but remain poorly investigated in the pseudomonads.

Amyloids consist of protein monomers, which upon self-assembly stack as β-strands perpendicular to the fibril axis in the so-called cross-β structure (Tycko 2004; Nelson et al. 2005). FuBA fibrillation is under close temporal and spatial control, avoiding potentially cytotoxic effects of prefibrillar intermediates common to unregulated amyloid formation (Gebbink et al. 2005; Krishnan and Lindquist 2005; Epstein and Chapman 2008; Dueholm et al. 2011). Despite the common ability to form amyloid fibrils, FuBA monomers from different organisms share little to no amino acid sequence similarity (Shewmaker et al. 2011). Another common property of FuBA is their insolubility and extreme mechanical and chemical stability, resisting dissolution by urea and boiling in sodium dodecyl sulfate (SDS). Consistent with a biologically optimized system, FuBA formation occurs over a wide range of aggregation conditions (Collinson et al. 1991, 1992; Wessels et al. 1991; Alteri et al. 2007; Dueholm et al. 2011). Noticeable features of FuBA also include auxiliary proteins insuring, for example, targeted deposition of monomers and the regulation of fibrillation (Gebbink et al. 2005; Epstein and Chapman 2008). FuBA may serve as surfactants, adhesins, biofilm structural components, spore coating and host cytotoxic compounds as well as combinations thereof (Olsén et al. 1989; Donlan 2001; Gebbink et al. 2005; Nielsen et al. 2011).

Several studies have shed more light on the molecular machineries required for FuBA expression (White et al. 2001; Elliot and Talbot 2004; Epstein and Chapman 2008). Curli fibrils from *Escherichia coli* and *Salmonella* remain the most well-characterized system, demonstrating the concerted expression of transport proteins, transcription factors, and fibrillation nucleators to produce the final amyloid fibril (Barnhart and Chapman 2006; Epstein and Chapman 2008; Otzen 2011; Taylor et al. 2011). Other well-studied examples of FuBA include chaplins from *Streptomycetes coelicolor*, the *Bacillus subtilis* protein TasA, and harpins of *Xanthomonas axonopodia* (Claessen et al. 2003; Oh et al. 2007; Romero et al. 2010).

In a recent study, the presence of a six-gene operon (*fapA-F*) responsible for expression of functional amyloid in *Pseudomonas* (Fap) in *P. fluorescens* UK4 (UK4) was discovered. Bioinformatics was used to demonstrate that homologous operons are present within several *Pseudomonas* species, including *P. aeruginosa*, *P. fluorescens*, and *P*. *putida* (Dueholm et al. 2010). *Pseudomonas aeruginosa* is an opportunistic human pathogen notoriously responsible for infectious biofilm in cystic fibrosis patients, chronic wounds, and on medical devices (Donlan 2001; Høiby 2006). Strains of *P. fluorescens* and *P. putida* are known plant growth-promoting bacteria, interacting with plant roots through, among other factors, secreted proteins and biofilm formation (Espinosa-Urgel et al. 2000; Haas and Défago 2005). Furthermore, strains of *P. putida* are prime candidates for bioremediation as they metabolize organic solvents and environmental toxins (Parales et al. 2000; Attaway and Schmidt 2002). The function of Fap and extent of expression in these pseudomonads remain currently unknown. The *fap* operon of UK4 enabled a laboratory *E. coli* strain to form biofilm, but other specific functions of *fap* and the individual Fap proteins remain unclear (Dueholm et al. 2010).

Given the medical and technical relevance of biofilm formation by the *fap* containing *Pseudomonas* species, we investigated the capacity of the three different strains, *P*. *aeruginosa* PAO1 (PAO1), *P. fluorescens* Pf-5 (Pf-5), and *P. putida* F1 (F1), to express amyloids, and the effect of amyloid expression on aggregation and biofilm formation. These strains do not express amyloids in a detectable quantity under typical laboratory growth conditions, which motivated our use of recombinant *Pseudomonas* cell lines to probe the effect of *fap* expression. We investigated the phenotypic effects on aggregation and biofilm formation to assay for functional importance, and analyzed the biophysical properties of the fibrils. As the *fap* operon seemingly encompassed all the genes necessary to form FuBA, we also used a bioinformatic approach to investigate the individual gene functions and thus uncover similarities between *fap* and the *E. coli* curli system (Barnhart and Chapman 2006).

## Experimental Procedures

### Bacteria and media

Growth medium for shake flask cultures was colony factor antigen (CFA) medium (10 g/L hydrolyzed casein, 50 mg/L MgSO_4_, 5 mg/L MnCl_2_, 1.5 g/L yeast extract, pH 7.4 in double distilled water). 40 mg/L tetracycline added for recombinant organisms. Incubation was at 25–37°C and 200 rpm. Bacteria strains and primers for PCR amplification of the *fap* operons were as listed in Table 1. Cloned plasmids (see below) contained the *Pseudomonas fap* operons controlled by an isopropyl β-D-1-thiogalactopyranoside (IPTG) inducible *lac*UV5 promoter. The *lac*-type promoters, however, are known to be leaky, but approximately threefold higher expression levels were obtained with IPTG induction.

**Table 1:** Bacteria, plasmids, and primers used in this study. The underlined primer sequences included restriction enzyme cleavage sites

Species and strain	Characteristics/Sequence	Reference
*Escherichia coli*		
Mach1	Used for routine subcloning	Invitrogen
INV110	Nonmethylating plasmid host	Invitrogen
S17-1	*pro thi recA hsdR (r*^*−*^ *m*^*+*^*)* Tp^r^ Sm^r^ Km^r^ Ω RP4-2-Tc::Mu-Km::Tn7	Simon et al. (1983)
DH5α	F^−^, φ80d*lac*ZΔ M15, Δ(*lacZYA-argF*)U169, *deoR*, *recA*1, *endA*1, *hsdR*17(rk^*−*^, mk^+^), *phoA*, *supE*44, λ^*−*^, *thi*-1, *gyrA*96, *relA*1	Invitrogen
HB101/pRK600	Sm^r^, *recA thi pro leu hsdRM*^+^ with pRK600	Klausen et al. (2003)
HB101/pUX-BF13	Sm^r^, *recA thi pro leu hsdRM*^+^ with pUX-BF13	Klausen et al. (2003)
HB101/pBK-mini-Tn7(Sm^r^)-gfp	Sm^r^, *recA thi pro leu hsdRM*^+^ with pBK-mini-Tn7(Sm^r^)-gfp	Klausen et al. (2003)
*Pseudomonas*		
*P. fluorescens* UK4	Wild type	Dueholm et al. (2010)
*P. fluorescens* UK4 pFap	UK4 with pMMB190Tc-UK4fap	This study
*P. aeruginosa* PAO1	Wild type	Jacobs et al. (2003)
*P. aeruginosa* PAO1 pFap	PAO1 with pMMB190Tc-PAO1fap	This study
*P. aeruginosa* PAO1 GFP	PAO1 tagged with eGfp in a mini-Tn7 construct; Sm^r^	This study
*P. aeruginosa* PAO1 Δ*fap*	*fapA-fapF* inactivated in PAO1 by allelic displacement with a gentamicin resistance cassette using pEX18Ap *fapA-fapF*; Gm^r^	This study
*P. aeruginosa* PAO1 Δ*fap* GFP	*fapA-fapF* inactivated in PAO1 by allelic displacement with a gentamicin resistance cassette using pEX18Ap *fapA-fapF*; Gm^r^; tagged with eGfp in a mini-Tn7 construct; Sm^r^	This study
*P. aeruginosa* PAO1 pV GFP	PAO1 with pMM190Tc; tagged with eGfp in a mini-Tn7 construct; Sm^r^	This study
*P. aeruginosa* PAO1 pFap GFP	PAO1 with pMM190TcPAO1fap; tagged with eGfp in a mini-Tn7 construct; Sm^r^	This study
*P. aeruginosa* PAO1 *fapA*::*lacZ*	PW4417, transposon mutant of PAO1 with *lacZ* introduced in-frame into *fapA*.	Jacobs et al. (2003)
*P. putida* F1	Wild type	ATCC 700007
*P. putida* F1 pFap	F1 with pMMB190Tc-F1fap	This study
*P. putida* F1 pFap (ΔFapA)	F1 with pMMB190Tc-F1fapΔA	This study
*P. fluorescens* Pf-5	Wild type	ATCC BAA-477
*P. fluorescens* Pf-5 pFap	Pf-5 with pMMB190Tc-Pf5-fap	This study
Plasmids		
pMMB190	IncQ *lacI*^q^ *bla*(Am^R^) *Ptaclac lacZα*	Morales et al. (1991)
pEX18Tc	Source of *tet*(Tc^R^)	Hoang et al. (1998)
pMMB190Tc	pMMB190 Δ*bla tet*(Tc^R^)	This study
pMMB190Tc-UK4fap	pMMB190Tc with UK4 *fap-A-F*	This study
pMMB190Tc-PAO1fap	pMMB190Tc with PAO1 *fap-A-F*	This study
pMMB190Tc-F1fap	pMMB190Tc with F1 *fap-A-F*	This study
pMMB190Tc-F1fapΔA	pMMB190Tc with F1 *fap-A-F* with premature stop codon in *fapA*	This study
pMMB190Tc-Pf-5fap	pMMB190Tc with Pf-5 *fap-A-F*	This study
pUX-BF13	*mob*^+^ori-R6K; helper plasmid providing the Tn7 transposition functions *in trans*; Amp^r^	Bao et al. (1991)
pBK-mini-Tn7(Sm^r^)-gfp	Delivery plasmid for mini-Tn7-P_A1/04/03_-gfp; Amp^r^, Sm^r^	Koch et al. (2001)
pRK600	*ori*-ColE1 RK2-*mob*^+^RK2-*tra*^+^ helper plasmid for conjugation; Cm^r^	Kessler et al. (1992)
pDONR221	Gateway donor vector; Km^r^	Invitrogen
pEX18ApGW	Gateway compatible gene replacement vector; Suc^s^, Amp^r^	Choi and Schweizer (2005)
pPS856	0.83 kb blunt-ended SacI fragment from pUCGM ligated into the EcoRV site of pPS854; Amp^r^, Gm^r^	Hoang et al. (1998)
pDONR221 *fapA-fapF*	*fapA-fapF* entry clone; Km^r^, Gm^r^	This study
pEX18Ap *fapA-fapF*	*fapA-fapF* knockout vector; Suc^s^, Amp^r^, Gm^r^	This study
Primers		
TetR-Fw	5'-CCTCGTGATACGCCTATTT	This study
TetR-Rw	5'-GCGGTGGTTTTTTTGTTTG	This study
EcoRI-UK4fapFw	5'-CACTGAATTCGCTTCTGCTCTATTCCTCAC	This study
HindIII-UK4fapRw	5'-CACTAAGCTTGCGCAGCGGTTTTAGAAGT	This study
EcoRI-PAO1fapFw	5'-CACTGAATTCCCCTGCCAACGCTGATTA	This study
HindIII-PAO1fapRw	5'-CACTAAGCTTGTGGCCCCTCAGAAGTAGT	This study
EcoRI-F1fapFw	5'-CACTGAATTCTTCTGCTCTGTCGTTCGC	This study
BamHI-F1fapRw	5'-CACTGGATCCGATGGCAACTATCAGAAGTA	This study
EcoRI-Pf-5fapFw	5'-CACTGAATTCGAAACAGTCCCGAAAGCC	This study
BamHI-Pf-5fapRw	5'-CACTGGATCCCGGTGGGTCAGAAGTAGT	This study

### Cloning of a tetracycline selective broad host expression vector

At the time this study was initiated, all public available broad host expression vectors used the ampicillin-resistant gene (*bla*) as selective marker. The strains investigated in this study, however, have natural resistance against this antibiotic. A modified version of the broad host expression vector pMMB190, in which a tetracycline resistance gene replaced the *bla* gene, was therefore constructed (pMMB190Tc). This was achieved by amplifying the tetracycline resistance gene from pEX18Tc by PCR using the TetR-Fw and TetR-Rw primer pair (Table 1). The PCR was performed using the Pfu DNA polymerase (Life technologies, Paisley, UK) in a standard reaction mixture as suggested by the manufacture and the following PCR settings: Initial activation (95°C, 180 sec), 25 cycles of denaturation (94°C, 30 sec), annealing (56°C, 60 sec), and extension (72°C, 180 sec) followed by a final extension (72°C, 10 min). pMMB190 was transformed into the nonmethylating *E. coli* strain INV110 (Life technologies) and purified using the Fast Plasmid kit (5 Prime, Hamburg, Germany). Purified plasmid was digested with FastDigest BsaI (Life technologies), trimmed with Klenow fragment (Life technologies), and dephosphorylated with shrimp alkaline phosphatase (Life technologies) according to the manufactures' recommendations. The tetracycline fragment was ligated into linearized plasmid using T4 DNA ligase (Life technologies) according to the manufactures' recommendations. The resulting vector was confirmed by shotgun sequencing of the whole plasmid (Macrogen, Seoul, South Korea).

### Cloning the *Pseudomonas fap* operons into pMMB190Tc

Whole *fap* operons from UK4, PAO1, Pf-5, and F1 were amplified by PCR using the primer sets shown in Table 1. PCR was carried out using AccuPrime Pfx polymerase (Life technologies) in a standard reaction mixture as suggested by the manufacturer with the following PCR settings: Initial activation (95°C, 120 sec), 25 cycles of denaturation (95°C, 30 sec), annealing (60°C, 60 sec), and extension (68°C, 6 min) followed by a final extension (68°C, 10 min). Following PCR, A-overhangs were added using a taq polymerase (Life technologies) according to the manufacturers' recommendations. The resulting fragments were gel purified and subcloned into pCR4-TOPO vectors (Life technologies). Fragments containing *fap* operons were obtained by digestion of the subcloned vectors with FastDigest EcoRI/BamHI (F1 and Pf-5) or FastDigest EcoRI/HindIII, (UK4 and PAO1) followed by gel purification using the UltraClean 15 DNA kit (MO-BIO, Carlsbad, CA). The pMMB190Tc was prepared for ligation using the same restriction enzyme combinations and purified as above. Ligations were done using T4 DNA ligase (Life technologies) according to the manufacturers' recommendation and the resulting vectors (pFap) were confirmed by shotgun sequencing of the whole plasmids (Macrogen).

### Transformation of pFap plasmids into homologous hosts

Electrocompetent *Pseudomonas* cells were prepared from overnight cultures grown in LB medium at 37°C (PAO1) or 30°C (UK4, Pf-5, and F1). For each transformation, bacteria were harvested from 6 mL culture (7500*g*, 10 min). The cells were washed twice in 4 mL of 300 mM room temperature sucrose before suspension in 100 μL of 300 mM sucrose. 100 μL of each strain was mixed with 5 μL of purified vector and 40 μL of the suspension was transferred to a 1-mm gap electroporation cuvette. Electroporation was performed with a MicroPulser electroporator (Biorad, Hercules, CA) using 1.80 kV (PAO1) or 1.25 kV (UK4, Pf-5, and F1) pulses. 1 mL super optimal broth (SOC) medium was applied directly after electroporation and the sample was transferred to a 15-mL tube and incubated (28°C, 200 rpm, 2 h). 100 μL of the transformations were plated on LB agar plates containing 50 μg/mL tetracycline and the plates were incubated at 37°C (PAO1) or 30°C (UK4, Pf-5, and F1) until visible colonies appeared (1–3 days).

### Construction of a *Pseudomonas* PAO1 Δ*fap* mutant

A knockout fragment containing a gentamicin (Gm) resistance cassette was generated by PCR overlap extension essentially as described by Choi and Schweizer (2005). Primers (whose sequence will be supplied upon request) were used to amplify chromosomal regions upstream and downstream of PAO1 *fapA*-*fapF*, and to amplify a Gm resistance cassette from plasmid pPS856 (Hoang et al. 1998). The PCR fragments were fused together and amplified with primers GW-attB1 and GW-attB2 incorporating the *att*B1 and *att*B2 recombination sites at either end of the knockout cassette. Using the Gateway cloning system (Life technologies), the resulting knockout fragment was first transferred by the BP reaction into pDONR221 generating entry plasmid pDONR221 *fapA*-*fapF*, and subsequently transferred by the LR reaction into pEX18ApGW generating the knockout plasmid pEX18A*fapA*-*fapF*.

A PAO1 *ΔfapA*-*fapF* mutant (PAO1 *Δfap*) was constructed as follows: The pEX18A*fapA*-*fapF* knockout plasmid was transferred into PAO1 by two-parental mating using the donor strain *E. coli* S17-1 with selection on *Pseudomonas* Isolation agar plates supplemented with Gm. Resolution of single crossover events was achieved by streaking on 5% sucrose plates via the counter-selectable *sacB* marker on the knockout plasmid. The mutant construction was confirmed by PCR analysis.

### Green fluorescent protein tagging of PAO1 strains

The PAO1 wild type and derivatives were green fluorescent protein (GFP) tagged by inserting a mini-Tn7-*gfp* cassette into a neutral site of the genome, using four-parental mating, essentially as described previously by (Klausen et al. 2003).

### *Pseudomonas aeruginosa* PAO1 *fap* promoter activity

Activity of the *P. aeruginosa* PAO1 *fap* promoter was determined using the FluoReporter lacZ/galactosidase quantification kit (F-2905, Life technologies) and the *fap* promoter reporter mutant PAO1 *fapA*::*lacZ*. Optical density at 600 nm (OD_600 nm_) was measured during growth and 1 mL culture samples were collected in triplicate. Bacteria was pelleted by centrifugation (14 000*g*, 1 min) and resuspended in 1 mL of enzymatic lysis buffer (10 mM Tris-HCl, 0.1 g/L DNase I (#DN25, Sigma-Aldrich, St. Louis, MO), 0.1 g/L RNase A (#83833, Sigma-Aldrich) 0.1 g/L alginate lyase (#A1603, Sigma-Aldrich), 1 g/L lysozyme (#L6876, Sigma-Aldrich), 1 mM MgSO_4_, 0.1% (V/V) triton-X100). The samples were subjected to three freeze–thaw cycles using a water bath at 37°C and a −80°C freezer. Between each cycle the samples were mixed using a vortex mixer and samples were kept in the freezer after the final cycle. β-galactosidase activity was measured in each sample according to the FluoReporter lacZ/galactosidase quantification kit recommendations and the results were normalized according to OD_600 nm_.

### Shake flask culturing and sampling

A volume of 5 mL CFA media in 50 mL centrifuge tubes were seeded with frozen glycerol stocks and grown overnight. A volume of 50 mL CFA medium in 250 mL shake flasks were inoculated to an initial OD_600 nm_ of 0.075 and grown until 0.5 before induction with a final concentration of 1 mM IPTG. One culture flask for each culture was used as reference, with the remaining incubated undisturbed. At an OD_600 nm_ of 1.5, cultures were harvested for biofilm assays, bright-field microscopy, and transmission electron microscopy (TEM). For purification of fibrils in bulk, expression was done in 400 mL cultures and 2.5L shake flasks. Harvest was done by centrifugation at 28 000*g* for 30 min. Pellets were resuspended in 8.75% (volume/culture volume) buffer (10 mM Tris-HCl at pH 8) and homogenized using a power drill mounted tissue grinder. A proportion of 1.25% (volume/culture volume) enzyme mix (0.4 g**/**L RNase A, 0.4 g**/**L DNase I, 4 g**/**L lysozyme, 4 mM MgCl_2,_ and 0.4% (V/V) Triton X-100) was added and following 0.5 h incubation at 37°C, harvests were frozen at −80°C.

### Crystal violet biofilm assay

Biofilm formation in microtiter plates was quantified by crystal violet staining essentially as described by O'Toole and Kolter (1998). A volume of 100 μL of LB, containing 1000-fold diluted overnight culture, was added to the wells of a 96-well microtiter plate (#92096, TPP, Trasadingen, Switzerland) #92096), which was subsequently incubated (37°C, 18 h, 175 rpm). The wells were aspirated and remaining planktonic bacteria were removed by addition and removal of 120 μL 0.9% saline. The biofilms were then stained for 15 min with 120 μL 0.1% crystal violet (Sigma-Aldrich) in 0.9% saline. Crystal violet quantification was done by washing the wells twice with 150 μL 0.9% saline, solubilizing the crystal violet with 96% ethanol for 30 min, and measuring the absorbance at 590 nm. For this assay, leaky expression of cloned *fap* operons was sufficient and no IPTG was used.

### Cultivation of flow-chamber biofilms

Biofilms were cultivated at 37°C in flow chambers which were assembled and prepared as described previously (Sternberg and Tolker-Nielsen 2006). Flow chambers were inoculated with *P. aeruginosa* overnight cultures diluted to an OD_600 nm_ of 0.01 in FAB-glucose medium as described by Pamp et al. (2008). Replicate experiments were done with the velocity of the laminar flow in the flow-chamber channels at 0.2 mm/sec, using a Watson Marlow 205S peristaltic pump (Watson Marlow, Falmouth, U.K.). For this cultivation, leaky expression of cloned *fap* operons was sufficient and no IPTG was used.

### Microscopy and image analysis

Microscopic observation and image acquisition of biofilms were performed with a Zeiss LSM 710 confocal laser scanning microscope (Carl Zeiss, Oberkochen, Germany) equipped with an argon laser and detectors and filter sets for simultaneous monitoring of GFP (excitation, 488 nm; emission, 517 nm). Images were obtained using a 63x/1.4 objective. Simulated fluorescence projections were generated using the IMARIS software package (Bitplane AG, Zürich, Switzerland).

### Amyloid fibril purification

Harvests were subjected to three cycles of thaw–freeze using a 37°C water bath (0.5 h) and a −80°C freezer (>1.5 h) followed by another 2 h incubation at 37°C. Preparations were boiled three times in 2% (W/V) SDS and subsequently washed two times in buffer (10 mM Tris-HCl at pH 8) before final resuspension. Insoluble material was collected by centrifugation 0.5 h at 28 000*g*. When washing away SDS during the last purification steps, the purified, insoluble materials became very sticky and adhered to laboratory plastics.

### Amyloid SDS-PAGE

A volume of 2 × 25–250 μL samples from each of the purified preparations were frozen in liquid nitrogen and lyophilized overnight. Samples were then resuspended in 50 μL formic acid (FA) or milliQ water, respectively, lyophilized again. Samples were finally resuspended in 50 μL reducing SDS-PAGE loading buffer (75 mM Tris, 0.6% (W/W) SDS, 15% (V/V) glycerol, 7.5% (V/V) β-mercaptoethanol, 0.9 mg/L bromophenol blue, 8 M urea at pH 6.8). Urea was required to avoid precipitation of amyloid proteins. A volume of 4–15 μL was loaded to an AnyKD gel (Biorad) and electrophoresis done at 200 V for 0.5 h. Gels were stained with Coomassie Brilliant Blue G250 and digitally scanned.

### Tandem MS peptide sequencing

SDS-PAGE gel protein bands were excised, split in two, and in-gel digested using trypsin or chymotrypsin with ProteaseMax detergent (Promega, Madison, WI). In situ digestion was done according to modified protocol of Shevchenko et al. (1996) including reduction and alkylation of Cys. The peptides were analyzed on a nanoflow UPLC (Dionex Ultimate3000/RSLC, ThermoFisher Scientific, Waltham, MA) system coupled online by a nanospray ion source (Proxeon, ThermoFisher Scientific) to an Orbitrap Q-Exactive mass spectrometer (ThermoFisher Scientific). The peptides were separated on two successive reverse phase columns (Acclaim PepMap100 C18 Nano-Trap, and Column Acclaim PepMap300 C18, ThermoFisher Scientific) using a linear gradient (10–35% acetonitrile in 35 min) and a constant flow rate of 300 nL/min. The mass spectrometer was operated in a data-dependent mode to switch between full MS scans and tandem MS/MS. Fragmentation was performed using higher energy collision induced dissociation (HCD) and sequenced precursor ions were dynamically excluded for 30 sec. The raw mass spectrometry files were analyzed and exported as mgf using Thermo Proteome Discover (version 1.3.0.339). MS/MS spectra were searched (Matrixscience Ltd., London, U.K., in-house Mascot server version 2.3) against Uniprot database (complete proteomes UK4; PAO1; Pf-5; F1) choosing carboxymethyl (C), propionamide (C), and oxidation (M) as variable modifications and allowing enzyme specificity at one terminus only (Perkins et al. 1999). Peptide and fragment tolerances were set at 10 ppm and 20 mmu, respectively.

### Fourier transformed infrared spectroscopy

Fourier transformed infrared (FTIR) was carried out using a Tensor 27 FTIR spectrophotometer (Bruker, The woodlands, TX) equipped with a deuterated tri-glycine sulfate Mid-infrared detector and a Golden Gate single reflection diamond attenuated total reflectance (ATR) cell (Specac, Kent, U.K.). Purified fibers from cultures were dried on the ATR crystal using dry nitrogen. ATR spectra were recorded from 4000–1000 cm^−1^ using a nominal resolution of 2 cm^−1^ and 64 accumulations. Resulting spectra were baseline corrected and interfering signals from H_2_O and CO_2_ were removed using the atmospheric compensation filter in the OPUS 5.5 system (Bruker). Different components of the amide I region were identified by second derivative analysis in the OPUS 5.5 system.

### Transmission electron microscopy

Suspensions culture samples and purified amyloid fibrils were mounted on 400 mesh carbon coated, glow discharged nickel grids for 30 sec. Grids were washed with one drop of double distilled water and stained with three drops of 1% (W/V) phosphotungstic acid at pH 7.2. Samples were inspected in a transmission electron microscope (JEM-1010, JEOL, Eching, Germany) at 60 kV. Images were obtained using an electron sensitive CCD camera (KeenView, Olympus, Center Valley, PA). For size determination, a standard grid size 264 nickel plate (462-nm grid) was used.

### Bioinformatics

Searching the NCBI Nucleotide collection (restricted to *Pseudomonas*, taxid:286*)* with the UK4 *fapC* sequence using BLASTn with NCBI default settings identified *Pseudomonas* harboring the *fap* operon. Hits were checked and confirmed to lie within a six-gene operon. Genomes from *Pseudomonas* hits were imported to CLC DNA Workbench 6.6.1 (CLCbio, Aarhus, Denmark) and genes translated using the standard genetic code translation table. Amino acid multiple sequence alignments (MSA) were made for each translated FapA-F homolog, respectively, using CLC run MUSCLE and the 90% conserved identity consensus sequence deduced from the alignments. FapB R1-R3 were cut from their origin sequences and combined to a single alignment to deduce the total repeat consensus sequence. Phylogenetic trees based on MSA were generated with the Neighbor Joining algorithm in CLC. To assay differences in evolution rates, MSA for the individual FapA-B, respectively, were used to generate pairwise comparison matrices in CLC. As a measure of total evolutionary differences, the half-sum (each pair appears twice) of all distances in the matrices were used. The same analysis was made for proteins from 88 curli csg operons. The curli operons were from *Enterobacteriales* retaining the *E. coli* operon organization: *Citrobacter* (3), *Cronobacter* (1)*, Enterobacter* (3), *Escherichia* (51), *Rahnella* (3), *Salmonella* (26), and *Shigella* (1). CLC distances were given as the Jukes–Cantor correction of the proportion between identical and overlapping alignment positions between the two compared sequences. To assay for presence of signal peptides, the FapA-F sequences were submitted to the SignalP 4.0 Server choosing gram-negative bacteria and allowing for transmembrane regions in the sequences (Petersen et al. 2011). Protein secondary and tertiary structures were assayed by submitting sequences to the Phyre2 server (http://www.sbg.bio.ic.ac.uk/phyre2) choosing the “intensive” option. Sequences were submitted without N-terminal signal peptides. Results were evaluated from the secondary structure predictions given in the result file and by investigating suggested 3D tertiary structures in the PyMOL Molecular Graphics System, Version 1.5.0.1 (Schrödinger, New York, NY).

## Results

### Assaying laboratory strains for *fap* expression

*Pseudomonas fluorescens* UK4 was originally identified as a Fap-producing strain through its ability to form deep-red colonies when grown on agar plates containing the amyloid-binding dye Congo red (Dueholm et al. 2010). However, despite extensive efforts, we have not been able to identify laboratory growth conditions under which PAO1, Pf-5, and F1 produce detectable amounts of Fap fibrils. For all organisms, shake flask growth in LB, tryptic soy broth, and CFA media at temperatures ranging from 25–37°C were assayed including variations in NaCl concentrations and culturing times (12–72 h) prior to purification. Finally, cultures spread on CFA and LB agar plates with harvest of culture layers were assayed. Presence of amyloid fibrils was investigated using TEM, Congo red, and amyloid conformational specific antibody (WO1) staining of bacteria cultures and amyloid purification (O'Nuallain and Wetzel 2002).

**Figure 1:**
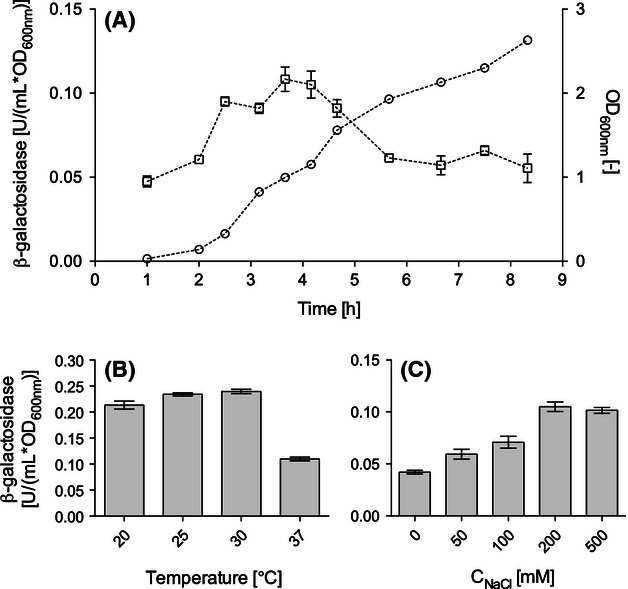
The *Pseudomonas aeruginosa* PAO1 *fap* operon is transcribed and transcription is affected by growth phase, temperature, and NaCl concentration. Promoter activity during growth was measured using the reporter strain PAO1 *fapA*::*lacZ*, which produced β-galactosidase under control of the *fap* promoter. β-galactosidase activity within samples was normalized according to OD_600 nm_. (A) Specific activity of the *fap* promoter during growth at 37°C in LB-medium containing 200 mM NaCl. Similar profiles were seen with other NaCl concentrations. β-galactosidase activity are marked by squares and OD_600 nm_ by circles. (B) Specific activity of the *fap* promoter in cultures grown in LB-medium at various temperatures. (C) Specific activity of the *fap* promoter in cultures grown in LB-medium containing various concentrations of NaCl. For B and C, the data show samples collected at OD_600 nm_ = 1.0–1.5.

To further investigate the expression of *fap* in wild type *Pseudomonas*, we employed a *P. aeruginosa* PAO1 strain with the β-galactosidase gene (*lacZ*) cloned chromo-somally in-frame into *fapA* (PAO1 *fapA*::*lacZ*). In this setting, PAO1 *fapA*::*lacZ* culturing allowed for measuring *fap* promoter activity at various growth conditions with a standard β-galactosidase activity assay. The activity measurements demonstrated that the *fap* promoter was constitutively active in shake flask cultures of PAO1 *fapA*::*lacZ* with a peak activity during the exponential growth phase (Fig. 1A). Furthermore, results also indicated that lower temperature and high salt concentrations favored promoter activity (Fig. 1B and C). No noteworthy phenotypical effects accompanied the promoter activity, however, an active promoter implied Fap expression in the wild type PAO1.

### Construction of homologous recombinant Fap model systems

In order to study the functional features of Fap expression, a recombinant approach was chosen where the *fap* operons from each strain, including UK4, were cloned into an inducible broad host expression vector, and reintroduced into the original strains for homologous Fap overexpression. This approach yielded four recombinant strains, termed UK4 pFap, PAO1 pFap, Pf-5 pFap, and F1 pFap. Fap fibrils can be purified from the UK4 wild type growing on agar plates, albeit not from suspension cultures (Dueholm et al. 2010). Unfortunately, suspension culturing for propagation of UK4 stocks reduces the yield of purified fibrils from agar plate culturing approximately 40-fold. However, the ability of UK4 to produce detectable amounts of Fap fibrils allows for comparisons of native and plasmid-encoded fibrils. In the case of PAO1, we also constructed a defined *fap* operon knockout mutant, and furthermore the PAO1 wild type, PAO1 pFap, and PAO1 Δ*fap* strains were chromosomally tagged with GFP. The more extensive experiments with PAO1 were motivated by its importance as a biofilm model system and a human pathogen (Harmsen et al. 2010).

### Expression of the *fap* operons results in aggregated growth and biofilm formation

**Figure 2:**
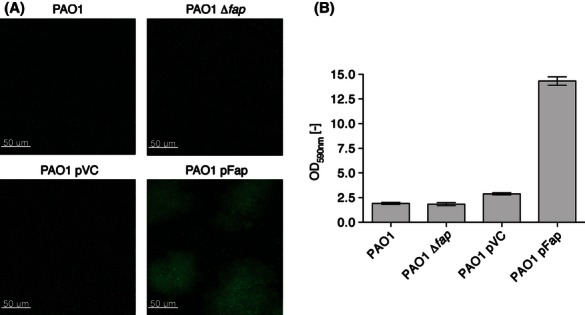
PAO1 wild type and derivatives cell aggregation and quantification of biofilm. (A) Confocal laser scanning micrographs of samples from GFP-tagged PAO1 shake flask cultures with images chosen to represent the average composition of samples. Bars, 50 μm. (B) Amount of biofilm formed in microtiter plates. The biofilm biomass was quantified by the use of a crystal violet staining assay. The mean and standard deviation of six replicates are shown.

The phenotypic effect of *fap* expression was first assayed in shake flask cultures, where the recombinant derivatives formed biofilm at the air-medium interface on flask sides. Cultures of the wild type strains and the recombinant counterparts were compared using confocal laser scanning microscopy (CLSM) (Fig. 2A). PAO1 pFap formed large clumps in shake flask cultures, whereas the PAO1 wild type and the vector control strain, PAO1 pVC, as well as the *fap* deletion mutant PAO1 Δ*fap* mainly, were present as planktonic cells. PAO1 pFap bacterial aggregates were also readily visible in the culture flasks and quickly settled from suspension. Expression of the *fap* operon thus led to a highly aggregative and adherent phenotype in liquid PAO1 cultures. Highly similar results were obtained with Pf-5, F1, UK4 wild type and their recombinant derivatives (data not shown).

Subsequently, the ability of the PAO1 strains to form biofilm in microtiter trays was investigated. The amount of biofilm formed after 24 h of incubation was quantified by the use of a crystal violet-based staining assay. PAO1 pFap formed five- to sixfold more biofilm than the PAO1 wild type and PAO1 pVC strain (Fig. 2B). Moreover, we found that PAO1 Δ*fap* formed biofilm to the same extent as the wild type under these conditions. The ability of *fap* overexpressing strains to form excessive amounts of biofilm was also demonstrated for Pf-5, F1, and UK4 (data not shown). We have made similar observations for *E. coli* overexpressing *fap* (Dueholm et al. 2010).

**Figure 3:**
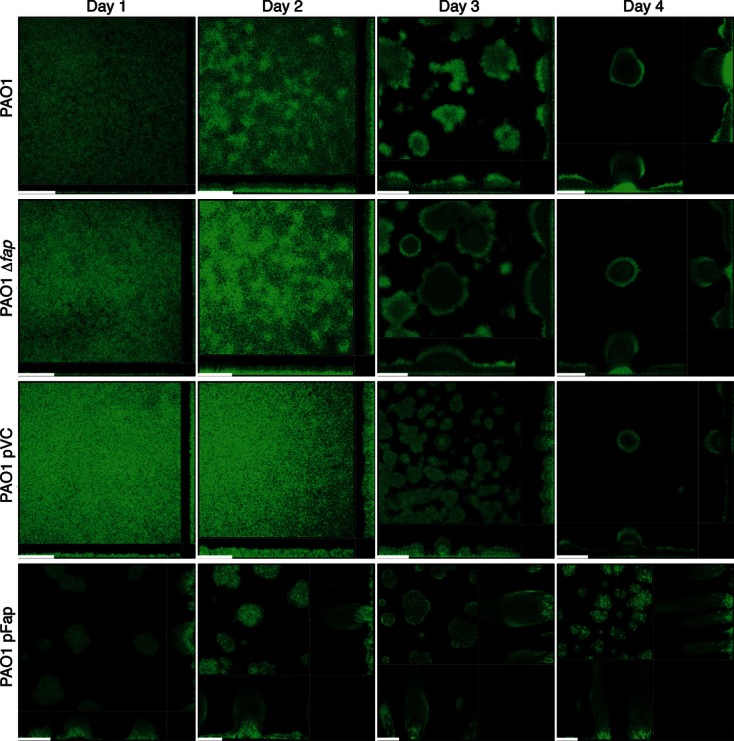
PAO1 biofilm formation in flow chambers. Confocal laser scanning micrographs of GFP-tagged PAO1 wild type and derivative cultures and biofilms formed in flow chambers. Images were captured after 1, 2, 3, and 4 days of cultivation. Each image contains top view (upper left) and two side view panes (bottom and right linings) with focus depth indicated (white markers). Bars, 50 μm.

The ability of the PAO1 strains to form biofilms was furthermore investigated using flow chambers. The PAO1 wild type, pVC, and Δ*fap* strains all attached to the glass surface (Day 1) and initially formed a flat biofilm (Day 2), followed by small microcolonies (Day 3), and eventually formed mushroom-shaped microcolonies (Day 4) (Fig. 3). The fact that the PAO1 Δ*fap* mutant formed biofilms similar to PAO1 wild type biofilms shows that Fap is not an absolute requirement for biofilm formation under these experimental conditions. The PAO1 pFap strain showed clear differences in biofilm formation and structure. Notably, PAO1 pFap initiated microcolony formation much earlier than the PAO1 wild type and pVC (Day 1), and after 4 days of cultivation the microcolonies had an elongated unusual appearance, presumably caused by the effect of shear force on these unusually large microcolonies (Fig. 3).

### Expression of the *fap* operons yield amyloid fibrils

**Figure 4:**
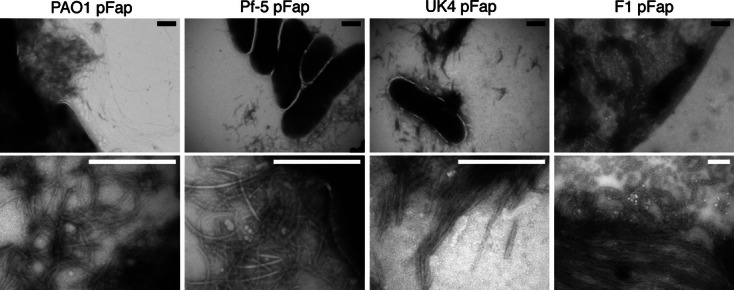
In situ imaging of Fap fibrils in recombinant cultures. Transmission electron microscopy (TEM) images of PAO1 pFap, Pf-5 pFap, UK4 pFap, and F1 pFap shake flask cultures at different magnifications. No fibrils were found in the corresponding wild type cultures (no images). Bars 0.5 μm.

The presence of amyloid fibrils in the *fap* overexpressing cultures was confirmed by TEM (Fig. 4). Copious amounts of fibrils were present both adjacent to bacterial cells and free in suspension. The fibrils varied slightly in morphology. PAO1 pFap and Pf-5 pFap fibrils were generally thicker and slightly more curved than the more straight and staggered ones of UK4 pFap. Fibrils from F1 pFap were somewhat intermediate between these two categories. The fibrils expressed by UK4 pFap were highly similar in morphology to those previously observed for UK4 wild type (Dueholm et al. 2010). As expected from efforts with Fap purification from wild type strains, no fibrils were seen in samples from the PAO1, Pf-5, F1, and UK4 wild type cultures. All the fibrils displayed a tendency to aggregate, and for UK4 pFap and F1 pFap the association was generally parallel with the length axis of the fibrils. Although the TEM sample preparation may influence the fibril association, this is unlikely to account for the consistent differences among the species.

**Figure 5:**
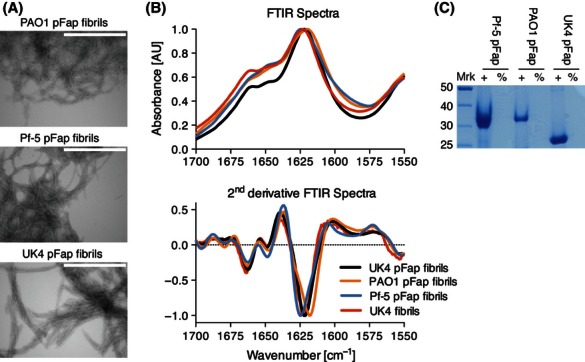
Analysis of purified fibrils. (A) TEM images showing fibrils identical to those in culture. (B) FTIR spectra (top) and with the calculated 2nd derivative spectra (bottom) for peak and shape identification. (C) SDS-PAGE gel of preparations resuspended in water (÷) or FA (+) prior to lyophilization and reconstitution in loading buffer. Calculated M_w_ of FapC is 33.0, 34.0 and 25.1 kDa for Pf-5, PAO1, and Pf-5, respectively.

Apart from the fibril shape, the hallmark of FuBA fibrils is their cross-β structure, aqueous insolubility, and stability, and UK4 Fap fibrils were previously confirmed to possess all these characteristics (Dueholm et al. 2010). Purification by repeated boiling in SDS demonstrated the fibrils' resistance to denaturing conditions. However, fibril purification for the F1 pFap culture failed, most likely because these fibrils were less stable than their counterparts and could not be efficiently separated from contaminants. The successfully purified preparations of PAO1, Pf-5, and UK4 Fap were also analyzed by TEM (Fig. 5A) and shown to contain fibrils with an identical appearance to those observed in the nonpurified culture samples.

To demonstrate the presence of amyloid structures, at the level of protein secondary structure, purified preparations were subjected to FTIR spectroscopy (Fig. 5B). The purified preparations all showed characteristic FTIR spectrum peaks (1617–1625 cm^−1^) well within the interval consistent with a cross-β amyloid structure (1615–1630 cm^−1^) (Zandomeneghi et al. 2004). The peaks at 1660–1662 cm^−1^ may indicate an antiparallel arrangement of the β-sheets, although this is not a definitive marker (López De La Paz et al. 2002; Zandomeneghi et al. 2004). Even more significant, the 2nd derivative FTIR spectra of PAO1 pFap and Pf-5 pFap fibrils were identical in shape with each other and with that of UK4 pFap fibrils. Furthermore, the three FTIR spectra of recombinant-expressed fibrils were identical in shape to that of Fap purified from the UK4 wild type in an earlier study (Dueholm et al. 2010), indicating the same structural constraints on their peptide backbone across all fibril species.

To determine the Fap fibrils monomer composition, purified preparations were subjected to reducing SDS-PAGE analysis with prior solubilization using pure FA (Fig. 5C). The SDS-PAGE analysis demonstrated that the proteinaceous contents of the preparations were composed of primarily one constituent each. Protein bands from the UK4, Pf-5, and PAO1 Fap preparations corresponded to the expected molecular weight (M_w_) of the FapC proteins from the respective organisms (UK4 ∼22.6, Pf-5 ∼30.7, and PAO1 ∼31.5 kDa). Gel bands (Fig. 5C) were confirmed to be mainly FapC by trypsin in-gel digestion followed by tandem mass spectrometry peptide sequencing. Interestingly, the excised gel bands were also all found to contain FapB (∼17 kDa) and FapE (∼23 kDa) although not consistent with the molecular weight expected from gel electrophoresis. However, given the sensitivity of the applied mass spectrometry instrument, any imperfections in the gel separation and contaminating proteins were detected. Notably, few high abundance membrane proteins, for example, OmpF- and LemA-like, were also identified, however, with significantly lower protein scores than the Fap proteins and also varying among samples. Protein bands corresponding to FapB and FapE were not detectable in the gels with intense bands of FapC (Fig. 5C), indicating that FapB and FapE only constitute a very small mass percentage of the purified fibrils.

### Manipulation of *fap* genes probes individual gene functions

**Figure 6:**
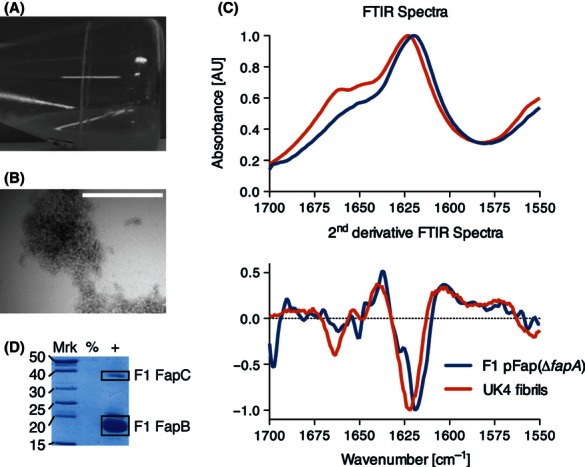
Analysis of the F1 pFap(Δ*fapA*) culture and purified fibrils. (A) Photograph of culture flask (turned on the side) showing a biofilm rim at the air-medium interface. (B) TEM images showing fibrils identical to those in culture. (C) FTIR spectra (top) and with the calculated 2nd derivative spectra (bottom) for peak and shape identification. (D) SDS-PAGE gel of preparations resuspended in water (÷) or FA (+) prior to lyophilization and reconstitution in loading buffer. The calculated M_w_ of the *Pseudomonas putida* F1 FapB and FapC are 19.9 kDa and 49.2 kDa, respectively. The faint band at ∼40 kDa was identified as FapB, likely migrating as a dimer.

The function of the protein FapA, the product of the first gene in the *fap* operon, remains currently unknown. However, a point mutation fortuitously created a stop codon in the middle of the *fapA* gene in a F1 pFap plasmid, and the phenotype of the resulting mutant (F1 pFap[Δ*fapA*]) indicated a potential function for FapA. F1 pFap(Δ*fapA*) showed extensive biofilm formation in shake flask cultures (Fig. 6A) albeit only minor aggregation of cells in suspension, while purification of the expected fibrils yielded short, fibril-like aggregates (Fig. 6B). The purified fibril-like aggregates produced a FTIR spectrum highly similar to that of the fibrils from an intact *fap* operon (compare Figs. 6C and 5B). SDS-PAGE analysis with subsequent mass spectrometry peptide sequencing (Fig. 6D) showed the copious amounts of insoluble, extremely stable aggregates produced by F1 pFap(Δ*fapA*) to consist of mainly FapB protein (∼18-kDa band, Fig. 6D) with small amounts of F1 FapC (∼38 kDa band, Fig. 6D). Apparently, FapB readily formed amyloid fibrils with only small amounts of FapC as the result of *fapA* deletion, and these also resulted in an aggregative and biofilm adhering phenotype. These results imply that FapA has a regulatory or facilitating function affecting the distribution of FapC and FapB in the final amyloid fibril.

### The Fap proteins are not equally conserved

**Figure 7:**
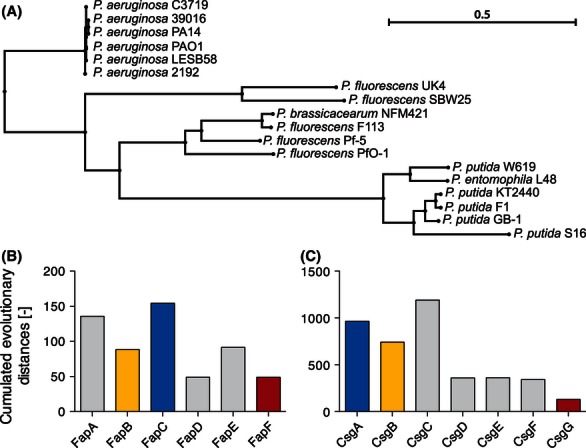
*Pseudomonas* FapC phylogeny and Fap evolutionary divergence. (A) FapC-based phylogenetic tree of 18 *Pseudomonas* harboring the *fap* operon. Scale bar indicates amino acid substitutions per position. (B) Measure of evolutionary divergence of the individual Fap proteins. Each bar indicates cumulated evolutionary distances for a given Fap protein across the 18 strains. This measure is equivalent to the summed branch lengths from a phylogenetic tree as presented in A. (C) Measure of evolutionary divergence of the individual curli Csg proteins across the 88 bacteria. Selected bars are colored according to predicted protein function: main amyloid monomer (blue), nucleator (yellow), and membrane pore (red) – see discussion.

The FapC protein of the UK4 *fap* operon was prior to this study the only verified *Pseudomonas* amyloid fibril monomer. An updated BLASTn search using the UK4 *fapC* to query the NCBI database identified 18 *Pseudomonas* strains that all harbored *fap* homologous operons. These strains were all found within the species *P. aeruginosa*, *P. brassicacearum*, *P. entomophila*, *P. fluorescens*, and *P. putida*. In all species, *fapC* was part of the six-gene *fap* operon (Fig. 7A). Examining the phylogeny of the strains using FapA-F showed these sequences had different degrees of conservation (Fig. 7B). FapA and FapC appeared less evolutionarily conserved than the most conserved proteins (FapD and FapF), while FapE and FapB showed an intermediate degree of conservation. An equivalent examination of the CsgBAC and CsgDEFG proteins from 88 *csg* curli operons from various *Enterobacteriales* (see methods) also demonstrated different degrees of conservation (Fig. 7C). In this case, CsgDEFG was more conserved than CsgA and CsgC with CsgB showing an intermediate degree of conservation.

### Structural and functional predictions of the Fap proteins

Assigning functions of the individual Fap proteins based on BLAST searches for homologs and motifs were unsuccessful. Protein tertiary structure prediction and homology analysis using the protein homology/analogy recognition engine (Phyre2), however, identified FapF as a β-barrel membrane pore and FapD as homologous with peptidase C39-like domains of various ABC transporters, that is, cysteine proteases. Purifications showed that FapB, FapC, and FapE were part of the extracellular amyloid fibrils, and a bioinformatic investigation found Type-I N-terminal secretion signal peptides in 104 of the 108 (6 × 18) Fap proteins and their homologs. The exceptions were *P. fluorescens* F113 FapA, *P. putida* S16 FapC and FapF, and *P. putida* W619 FapA. These exceptions are likely attributed to annotation errors. A separate study has previously found secretion signal peptides present in all proteins today known as Fap proteins from *P. aeruginosa* PAO1 (Lewenza et al. 2005). Amino acid sequence alignments of Fap proteins, not including secretion signal peptides, identified characteristic 100% conserved Cys residues. FapC from *P. brassicacearum, P. fluorescens* and *P. aeruginosa* organisms contained a C-terminal Cys-X-X-Cys motif and their FapE also had a Cys residue at the C-terminal. In contrast, *P*. *entomophila* and *P. putida* sequences did not contain these Cys motifs. All 18 *Pseudomonas* FapD sequences contained a Cys residue at 25 residues from their mature N-terminal. *Pseudomonas* FapA, FapB, and FapF sequences did not contain Cys residues outside the secretion signal peptides.

**Figure 8:**
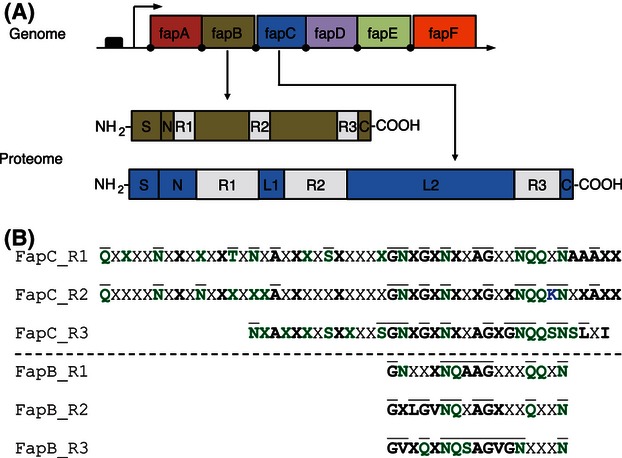
*fap* operon and FapB-C schematic overview. (A) Overview of the *fap* operon and below the FapB and FapC region structure. Widths of elements reflect their amino acid lengths. S: signal peptide, N: N-terminal region, R: repeat region, L: linker region, C: C-terminal region. (B) Repeat region consensus sequences from alignment of the 18 FapC and FapB protein sequences, respectively. Consensus cutoff was set at 90% identity across sequences. Overlined residue indicates 100% conservation. Residue in bold indicates >90% conservation of chemical properties: apolar (black), polar (green), and charged (blue). No formatting for residues with property conservation <90%.

We previously reported FapC to contain three imperfect repeat regions (R1-3) interspaced by so-called linker regions (L1-2), and also FapB was found to contain three similar repeat sequences (Fig. 8). An updated amino acid sequence alignment of all FapC and FapB homologs, respectively, identified novel properties of these repeats. Immediately N-terminal to the previously established FapC R1 and R2 sequences, we observed repeating sequences where Asn and Gln residues were 100% conserved across the 18 strains, indicating FapC R1 and R2 to be expanded versions of R3 (Fig. 8B). Repeat regions in FapB were all highly similar and truncated versions of those found in FapC (Fig. 8B). All the FapB and FapC repeat motifs showed characteristic conservation of Gln, Asn, and Ser residue rich stretches interrupted by Gly and/or Ala residues. A similar pattern was observed in the CsgA amyloid monomer of *E. coli* curli fibrils and shown to be important for amyloid fibril formation and stability (Wang and Chapman 2008). Despite sharing motif characteristics, FapB and FapC repeats were not homologous to those of CsgA.

**Figure 9:**
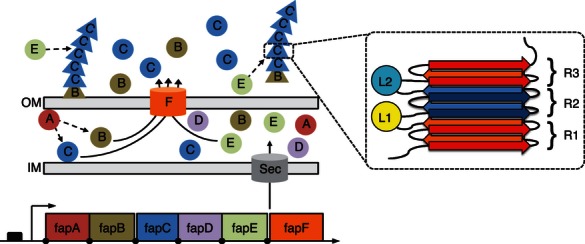
Conceptual models of the Fap molecular mechanism and the FapB/FapC amyloid fibril. (Left) Proposed functions of the Fap proteins. All Fap proteins are secreted via Sec across the inner cell membrane (IM). FapB, FapC, and FapE are further secreted across the outer cell membrane (OM) via FapF, where FapB nucleates fibrillation of FapC and FapE associates with the final fibril. Fibrils free in culture suspension were observed as well as adjacent to cells. FapD and FapA may reside in the periplasm, where FapA in an unknown manner affects FapB and FapC incorporation into the final fibril, for example, via their secretion, while FapD is likely a protease involved in proteolysis of Fap proteins. (Right) Suggested alignment of FapB and FapC protein regions in the mature FuBA. The linker regions may form additional β-strands inside the amyloid core, thus the position of the linker regions may deviate from the presented model.

Repeat motifs are expected to make up the β-strands of the amyloid fibril cross-β structure. The length and key conservation of Gly residues implied each repeat folded to form several β-strands. To probe FapB and FapC folding, protein sequences were submitted to Phyre2 for secondary and tertiary structure predictions. In general, tertiary structure predictions were limited due to the lack of determined structures for any homologs. FapB secondary structure predictions, however, indicated R1-3 consisted of two β-strands each with a possible turn centered on the 100% conserved Ala–Gly residues roughly in the middle of the repeat regions. Predictions also indicated that FapB contained α-helical segments outside the repeat regions. The tertiary structure predictions could not fully accommodate the predicted strands, although FapB R1-2 showed a tendency to fold in a strand-loop-strand-like motif. Secondary structure predictions for FapC showed extensive β-strands throughout the sequence. Given that the FapB repeats were truncated versions of the FapC R1-3, the results suggested FapC R1 and R2 could each form approximately four β-strands and R3 three β-strands (Fig. 9).

## Discussion

### Fap fibrils enhance cell aggregation, attachment, and provide strength to biofilms

Recombinant expression of the *fap* operons in *P. aeruginosa*, *P. fluorescens*, and *P. putida* suspension cultures had a dramatic effect on their mode of growth. The otherwise planktonic cells turned highly aggregative and formed extensive biofilms in solution, on the culturing flask at the air-medium interface, and in the microtiter assay. Furthermore, very early microcolony formation and extremely large biofilm structures were observed for PAO1 pFap in the flow chamber. To our knowledge, such unusually large structures have never been observed before in PAO1 flow-chamber biofilms, and it highlights the potent effect of Fap on PAO1 biofilm formation. Collectively these results show that the *fap* formed fibrils likely function as an adhesin for the attachment to an abiotic surface, and also as a stabilizing structural component providing strength to the mature biofilms. Interestingly, the purified fibril material also adhered vigorously to laboratory plastic and glass surfaces (see methods), supporting an adhesive function. The results, however, also showed that the Fap fibrils were not an absolute requirement for adhesion and biofilm formation under the tested culturing conditions tested. The PAO1 Δ*fap* mutant did not show any biofilm deficiency compared with PAO1 wild type, likely because biofilm formation in microtiter and flow-chamber studies also involves other components, for example, pili, eDNA, and polysaccharides (Harmsen et al. 2010). In this context, Fap expression greatly added to the PAO1 biofilm-forming capacity. The question remains, why it was not possible to show any significant expression of the amyloids in the wild type strains at the protein level in spite of the *fap* promoter activity, except for the agar plate grown UK4 (Dueholm et al. 2010). A likely explanation is that the PAO1 cultures do not express *fap* in significant amounts. A low expression level may be a laboratory artifact and if so, would also explain the lack of differences in biofilm properties of the Δ*fap* mutants and the wild types.

### Laboratory growth conditions apply selection pressure against Fap expression

It is likely that wild type strains lose the ability to express amyloids as laboratory growth conditions favor a planktonic lifestyle. This hypothesis is further supported by the significant decrease in the yield of Fap purified from our current stocks of UK4 wild type compared to the original isolate. Loss/modification of genes is not uncommon in lab cultures and even culture collection stocks of *P. aeruginosa* PAO1 undergo genomic diversification (Klockgether et al. 2010). Rainey et al. demonstrated the adaptive radiations of *P. fluorescens* SBW25 as a response to the heterogeneous growth conditions obtained in static cultures. This yielded three distinct morphotypes, the smooth morphotype (SM), the winkly-spreader (WS), and the fuzzy-spreader (FS). The SM is associated with planktonic growth, whereas the WS forms biofilm with cells adhering to surfaces and each other (Rainey and Travisano 1998). The FS is not very well described, but it also forms aggregates, albeit in the low oxygen zone near the bottom of the static culture. It was furthermore shown that homologous culturing applied a strong selection pressure for the SM. If Fap expression is associated with either the WS or the FS, this explains why the wild type bacteria did not express detectable amounts of Fap. Conversely, the domesticated, nonbiofilm forming *E. coli* K-12 has been shown to produce an OmpR-mutated variant, capable of curli expression, surface adherence, and aggregation, using atypical culturing conditions (Vidal et al. 1998).

### *Pseudomonas aeruginosa* and *P. fluorescens* Fap fibrils are FuBA composed of FapC with some FapB and FapE

The high thermal and chemical stabilities of the Pf-5, PAO1, and UK4 fibrils were demonstrated. The fibrils survived repeated boiling in SDS and required FA pretreatment to dissolve in loading buffer with 8 M urea. Protein assays showed fibrils from UK4 pFap, Pf-5 pFap, and PAO1 pFap consisted of mainly FapC protein, although with very small amounts of FapB and FapE included. This supported the hypothesis of FapB as an amyloid fibril component and potentially a fibrillation nucleator, also in agreement with the repeat homology of FapB and FapC. The role of FapE in the amyloid fibrils is more uncertain, although it may be speculated that the C-terminal conserved Cys residues of *P. fluorescens* and *P. aeruginosa* FapC and FapE, respectively, could be a site of protein–protein interaction. The FTIR spectra of purified fibrils were highly similar and consistent with recombinant fibrils having the amyloid cross-β structure. The FTIR spectra of the recombinant produced UK4 Fap were identical to those from the UK4 wild type, and the UK4 wild type fibrils were previously assayed by x-ray diffraction for confirmation of cross-β structure (Dueholm et al. 2010). Consequently, the FTIR data in this study indicated that fibrils from all species formed cross-β structures with similar constraints on the protein backbone, which also fits well with their similar repeat sequences forming the β-strands.

Regardless of multiple attempts we were not able to purify fibrils from F1 pFap, although Fap fibrils clearly were present in the cultures and conferred the same phenotypic effects as seen, for example, PAO1 pFap. Furthermore, the repeat sequences are highly similar to those of the other pseudomonads, that is, F1 fibrils may be an example of FuBA in terms of cross-β structure, while not displaying their classical high stability. The *B. subtilis* TasA protein also forms functional amyloids of lower stability. TasA fibrils will dissolve in 10% (vol/vol) FA or SDS-PAGE loading buffer, while fibrils purified for this study required > 90% (vol/vol) FA and withstood boiling in 2% (weight/vol) SDS (Romero et al. 2010). Compared to the homologs, F1 FapC lacks the C-terminal Cys-X-X-Cys motif. These Cys residues may engage in intermolecular disulfide bonds, and their absence may explain the putative decreased stability of the F1 Fap fibrils. In addition, F1 FapC also has an enlarged, hydrophilic L2 (∼260 aa), compared to the other strains (∼100 aa), and this could also affect stability.

### Operon manipulation provides information on the individual Fap proteins

The serendipitous stop codon in the F1 pFap(Δ*fapA*) allowed us to manipulate components of the vector-based *fap* operons to probe single gene functions and properties. Culturing and purification results from the F1 pFap(Δ*fapA*) showed that F1 FapB, with a small amount of F1 FapC, formed amyloid fibrils with a phenotypic effect similar to the overexpression of the intact *fap* operons. These observations further support the role of FapB as an amyloid protein and potential nucleator. The effect of interrupting *fapA* suggests that FapA is a chaperone for the amyloid monomers affecting the ratio of FapB to FapC in the final fibril. However, the specific interaction between FapC, FapB, and FapA remains unclear.

### The Fap operon shares common properties with the curli system

The homologous UK4, PAO1, Pf-5, and F1 *fap* operons conferred the ability to form FuBA, implying that the *fap* operon encompasses the necessary components for producing functional amyloids. In this sense, the *fapA-F* function is analogous to the *E. coli csg* operons, *csgBAC* and *csgDEFG* (Barnhart and Chapman 2006). The seven genes of the *csg* operons include a primary and nucleating amyloid monomer, CsgA and CsgB, respectively, a membrane pore (CsgG) and regulatory proteins (CsgC, CsgE, and CsgF). The combined results from culturing, biophysical and bioinformatic investigations of the *fapA-F* expression suggested parallels in FapA-F. A model sketching out a possible fibrillation machinery is provided in Figure 9.

FapA is potentially a chaperone for the amyloid monomers, while FapC and FapB are the main and nucleating amyloid monomers, respectively. The FapF β-barrel is a likely candidate for an outer membrane pore for FapB and FapC secretion to the extracellular environment. In keeping with this hypothesis, FapF is shown to be membrane associated in a proteome study of *P. putida-Cd001* isolated from *Arabidopsis halleri* rhizosphere (Manara et al. 2012). FapD could have proteolytic activity relevant for processing the Fap proteins during protein secretion, potentially functioning in combination with FapF. Further operon similarities include the targeting of FapA-F for Sec-dependent secretion to the periplasm, equivalent to the secretion of CsgE-F and CsgA-C (Barnhart and Chapman 2006). In addition, the FapC and FapB repeat regions' variation over the same motif of conserved Gln, Asn, and Ser residues is similar to that of CsgA. Phyre2 analysis indicated that repeats fold to multiple β-sheets in a strand-loop-strand-like motif also similar to the models proposed for the CsgA repeats in the mature fibril (Barnhart and Chapman 2006) (Fig. 9). The *fap* operons do not include a transcription factor equivalent to CsgD. However, organization of the *fap* genes into a single operon removes the need for an internal transcription factor.

Differences in FapA-F evolutionary divergence may support the functions suggested for Fap (compare Fig. 7B and C). Regulation of FuBA fibrillation, for example, to avoid cytotoxic intermediates, is a common feature across *Pseudomonas* strains, that is, regulatory proteins are subject to similar selection pressure. As an example, the function and outer membrane interaction of the FapF membrane is the same in the strains. Conversely, the extracellular fibril components are expected to be optimized for different habitats and growth conditions of the strains and thus subject to less stringent selection pressure. This hypothesis is further supported by the equivalent pattern observed for the CsgDEFG fibrillation regulating proteins relative to the CsgBA amyloid fibril monomers (Fig. 7C).

### Biological significance of the Fap operon

As *fap* is directly involved in *Pseudomonas* biofilm formation, Fap is potentially a virulence factor for *P. aeruginosa*. This is supported by the identification of a *fapC* deletion mutant of *P. aeruginosa* TBCF10839 as one of the most attenuated mutants, among 480 random transposon deletion mutants, in a *Caenorhabditis elegans* infection model and in a polymorphonuclear neutrophil leukocytes phagocytosis assay (Wiehlmann et al. 2007). Furthermore, investigations of the *E. coli* equivalent curli fibrils have also implicated these as a virulence factor (Collinson et al. 1992; Wang and Chapman 2008). In the case of *P. fluorescens* and *P. putida*, Fap fibrils are likely to be important in colonization of the rhizosphere. *P. fluorescens* forms biofilm-like colonies in grooves between plant root epidermal cells, while *P. putida* extracellular proteins are known to be important for adhesion to plant seeds in plant growth-promoting bacteria formulations (Espinosa-Urgel et al. 2000; Haas and Défago 2005). This is also consistent with *P. putida* biofilm, in general, having a high content of extracellular protein and FapF found in environmental rhizosphere samples (Jahn et al. 1999; Manara et al. 2012).

Based on the results of this study, we propose that Fap are extracellular biofilm components of equal importance to polysaccharides, other proteins, and eDNA. This hypothesis is also in agreement with the widespread presence of FuBA in nature (Larsen et al. 2007, 2008; Jordal et al. 2009). We further suggest that the *fap* operon comprises a molecular machinery for the spatially and temporally regulated formation of FuBA.
